# The Present Position and Some Resulting Troubles

**Published:** 1918-10-05

**Authors:** 


### THE INDIAN MEDICAL SERVICE.*

### The Present Position and Some Resulting Troubles.

The I.M.S., as the R.A.M.C. was, is restless because the members are not satisfied with their position and their pay. It is probable that position is more important in the matter than the pay, for the pay is not bad after a young officer has got away from Army work and into Civil. This nearly all try to do. In Civil there is pay of rank and pay of the billets held, for a man may be a pluralist, and usually pickings from private practice. A Civil surgeon in a large station often makes a considerable income from private practice, though the emoluments from this source have been greatly lessened by interference of the Indian Government with professional fees and honoraria, action that has caused great discontent in the I.M.S., which regards the regulations as unnecessary and insulting. The man with a regiment and the man in many small Civil stations is not well paid, but he has an ever-present hope to escape to Civil posts or better places. If it were not so, and a man had always to depend on the pay of his rank alone, the I.M.S. would have collapsed long ago, or been reformed.

Lack of position is a trouble. The regimental doctor is of no importance in the regiment. He is usually addressed as "Doc." or "Pills" even by officers many years junior to himself in age and some in service. He usually runs the mess and does odd jobs, and if he is nice and domestic and doesn't put on airs he is treated kindly and condescendingly. He finds it pays to know his place. Rather naturally, young men of parts and spirit change to "Civil" as soon as they can.

But when he changes to Civil he finds again that he is a person of little importance. All the I.C.S. is above him. He is under the Commissioner, under the Collector, and even under the "Stunt Sahib," if not officially, at least in common estimation. His precedence is his Army rank, but he has no comrade to support him, and a major is a nobody to an Indian civilian. He finds the need to be compliant to his juniors in years and his inferiors in parts. He is often actually under the orders of Indian civilians younger than himself, and, in any case, if consulted on official matters, it is solely on a health question, and his opinion is not of much weight. He has little or no command; an apothecary, a few gaolers, and so forth, are his underlings.

It is outside official duties and as a private practitioner that he recovers himself and his self-respect. Some of the finest operators, some of the wisest physicians have been in, and have come out of, the I.M.S. A man keen on his work, much on his own—has the expression ceased to be slang?—amongst a teeming population with a high percentage of disease, has unlimited opportunity for practice and for perfecting himself in any branch. Often his work brings good pecuniary reward, for fees are high to paying patients, but, as stated before, Government interference has greatly reduced the earnings. If he were paid at a similar rate for all he does in charity he would be a millionaire.

Another thing that is disturbing the I.M.S. is colour prejudice. Britons are conservative. The old dislike to being in a subordinate position to an Asiatic of low caste and no family still holds the field. To serve a native prince, to be on friendly terms with a native gentleman of rank and good repute, is one thing. To be ordered by a bazaar-bred buniah's child, or a babu's outcaste son, is another. These curious old primitive survivals flourish in the languorous East, they even live in the sturdy democratic West. The I.M.S. man, perhaps because he grows infected without knowing it by the native feeling round him, hates to have a native over him. It is possibly wrong, and is not to be supported by logic or quotations from progressive writers, but it is so. Time and better education may remove in the future these British prejudices, but till removed they must be taken into account.

It is hard to understand why Mr. Montagu thinks friction is inherent in the co-existence of the I.M.S. and the R.A.M.C. in India. The Services scarcely touch. The I.M.S. looks after native soldiery, certain other natives, and Indian civilians of all sorts. The R.A.M.C. looks after white troops, officers and men, their wives and children. The R.A.M.C. man knows he is a bird of passage. The I.M.S. man knows he is a fixture till death or a pension comes. It is true some of the Commands have an I.M.S. P.M.O., but this is not contact of a sort to cause friction to any extent. Promotion to these posts is not always welcomed, for often it means to the man selected the loss of an excellent and remunerative private practice, the break-up of an established home, and the exchange of work in which he is highly considered, happy, and interested for military administrative work of which he has no experience and the uncomfortable job of trying in his later years to direct the work of men who know far more about that work than the new director. If he should try to make himself felt, he probably will make unfortunate and ridiculous mistakes; if he leaves the work to his more experienced subordinates, he feels he is a nonentity. Neither position is satisfactory. We always come to position.

* See "The Hospital," June 8, p. 196.

(Continued on page 12.)

### THE INDIAN MEDICAL SERVICE.

(*Continued from page 8.*)

The discontents touched on have already had their effect on the entries for the I.M.S. It was the prize of State Medical Services. Now it is a long way second to the R.A.M.C. in the desire of the majority of the young medical men seeking careers. It is a sad thing for those who have spent the best of their years in what was the greatest Medical Service in the world to see it cankering to decay. Can a remedy be found? Can means be found to make the Service again attractive to the best of the schools?

THE HOSPITAL article of June 8 mentions a "proposal not new," that the R.A.M.C. should take over the Indian Army work, and seems to anticipate advantage from such a course. Would this be welcome to the R.A.M.C.? It would be interesting to see the proposals in a definite form. And what would become of the Indian Medical Service?

